# Effect of calcium ionophore on unfertilized oocytes after ICSI cycles

**Published:** 2012-03

**Authors:** Maryam Eftekhar, Farnaz Mohammadian, Fariba Yousefnejad, Parisa Khani, Abbas Aflatoonian

**Affiliations:** 1 *Research and Clinical Center for Infertility, Shahid Sadoughi University of Medical Sciences, Yazd, Iran.*; 2 *Department of Obstetrics and Gynecology, Zanjan University of Medical Sciences, Zanjan, Iran.*

**Keywords:** *Calcium ionophore*, *Unfertilized oocytes*, *ICSI cycles*

## Abstract

**Background:** Fertilization failure is one of the most problems in assisted reproduction technology (ART).

**Objective: **The aim of this study was the evaluation of oocytes activation by addition of calcium ionophore in unfertilized oocytes in ICSI cycles.

**Materials and Methods:** This study was done on 15 ICSI cycles (stimulated with standard long protocol). Mature retrieved oocytes with normal morphology that had no evidence of fertilization 24 hours after ICSI were included in the study. The oocytes with fertilization and unfertilized oocytes with degeneration were excluded from the study. The unfertilized oocytes were washed with GIVF medium and were transferred to GIVF medium that contained 5 µmol of calcium ionophore and were incubated for 10 minutes. Then again oocytes were washed with GIVF medium and consequently were transferred to GIVF medium and were incubated at 37°C in 6% CO_2_. After 18 hours, the oocytes were examined and activated oocytes were defined with observation of at least one pronucleus or cleaved oocytes.

**Results:** After ovarian stimulation and oocytes retrieval, 175 mature oocytes were obtained and injection of sperm was done for all of them. 114 of 175 oocytes (66%) showed evidence of fertilization after 24 hours. A total of 61 oocytes (34%) showed no evidence of fertilization and 10 oocytes were degenerated and were excluded from the study. Only 51 unfertilized oocytes with normal morphology were selected and were exposed to calcium ionophore. 37 (72.5%) of treated oocytes were fertilized (2PN) and 32 (62.7%) of them showed evidence of cleavage. 6 (11.8%) embryos had good quality.

**Conclusion:** According to our results, oocytes activation with calcium ionophore had an acceptable fertilization rate, however high quality embryos remained low. We propose future studies to evaluate embryo quality.

## Introduction

Fertilization failure is the most problem in assisted reproduction technology (ART). The main causes of fertilization failure in conventional IVF are extremely low counts, impaired motility, and abnormal morphology of sperm. Intracytoplasmic sperm injection (ICSI) was a revolution in ART and it is now the ultimate option to treat severe male factor infertility. During this procedure, sperm is directly inseminated into the oocyte (1-4). The average fertilization rate is 60-70% and total fertilization failure still occurs in 2-3% of ICSI cycles (1, 5-6). 

It reveals that fertilization failure after ICSI may be explained by defects in oocyte, sperm, or ICSI procedure. Both sperm and oocyte factors are believed to be involved in failed oocyte activation after ICSI (1, 5, 7). Oocyte activation is an early event in fertilization. It caused by material in the sperm that is thought to induce calcium release from the endoplasmic reticulum and also from other structures of the oocyte. Cytologic analysis of failed fertilization of human oocytes after ICSI showed that more than half of the failed fertilized oocytes are due to oocyte activation failure (8).

Various chemical substances have been identified that can induce calcium increase and oocyte activation, such as calcium ionophore, ethanol, ionomycin, puromycin, strontium chloride, probol ester and thimerosal. However, the use of these agents for artificial oocyte activation has been mostly limited to animal studies and case reports in human study (1, 9-15). In our study, we evaluated oocyte activation by addition of calcium ionophore in failed fertilized oocytes in ICSI cycles.

## Materials and methods

This experimental study was done from January 2008 to July 2009 in Yazd Research and Clinical Center for Infertility affiliated to Shahid Sadoughi University of Medical Sciences on 15 ICSI cycles. The trial design was approved by the Ethics Committee and all couples were required to sign a written consent before initiation of the treatment cycles. The couples who were candidate for ICSI (severe oligospermia, obstructive azospermia) were included in the study. The women with basal FSH >12 IU/ml, age >38 years, history of ovarian surgery or endocrine disorders were excluded from the study. 

Protocol of induction ovulation in all of patients was standard long protocol. Pituitary desensitization with a GnRH agonist and ovarian stimulation with gonadotropins (Merional, IBSA, Lugano, Switzerland), Sereno, were carried out. The patients monitoring was done by transvaginal ultrasound and hCG was injected when at least 3 follicles more than 18 mm in diameter were seen in ultrasonography. Oocytes were recovered transvaginally under ultrasound guidance and ICSI was done.

Mature oocytes with normal morphology, which had no evidence of fertilization 24 hours after ICSI were selected for this study and degenerated oocytes were excluded. The oocytes were washed with GIVF medium (Vitrolife- Sweden) and were transferred to GIVF medium that contain 5 µmol of calcium ionophore (A23187) (Sigma, St. Louis, Mo, USA) and were incubated for 10 minutes, then again oocytes were washed with GIVF medium and consequently were transferred to GIVF medium and were incubated at 37°C in 6%. After 18 hours the oocytes were examined and activated oocytes were defined with observation of at least one pronucleus or cleaved oocytes.

## Results

15 cycles of ICSI were included in our study. Basic and demographic characteristics of patients are showed in table I. After ovarian stimulation, 175 mature oocytes were obtained that injection of sperm was done for all of them. 114 of 175 oocytes (66%) showed evidence of fertilization after 24 hours. A total of 61 oocytes (34%) showed no evidence of fertilization. 10 oocytes were degenerated and excluded from our study and 51 unfertilized oocytes with normal morphology were selected and were exposed to calcium ionophore. 37 (72.5%) of treated oocytes were fertilized (2PN) and 32 (62.7%) of them showed evidence of cleavage. 6 (11.8%) of embryos had good quality on day 3 after addition of calcium ionophore (Table II).

**Table I:** Basic and demographic characteristics of patients

	**Mean±SD (n=15)**
Age of female (years)	28.53±4.20
Duration of infertility (years)	24.26±3.10
Basal FSH (IU/L)	6.2±3.21
BMI (kg/m^2^)	5.46±1.30

**Table II:** Activation of human unfertilized oocytes after ICSI by calcium ionophore

	**Number (%)**
Treated oocytes	51 (100)
Fertilized oocytes (2PN)	37 (72.5)
Cleaved oocytes	32 (62.7)
Good quality embryo	6 (11.8)

## Discussion

Despite of ICSI procedure improvement, fertilization failure still occurs. Fertilization failure in some of ICSI procedures are resulted from failed oocyte activation (2-3, 5). Artificial activation can be induced by several chemical agents. Calcium ionophore in intracytoplasmic sperm injection cycles has been effective in increasing fertilization rate (16). 

The present study was done on unfertilized oocytes after ICSI and approximately 72.5% of unfertilized oocytes extruded a second polar body within 24 hours after treatment with calcium ionophore. During second 24 hours incubation after activation, 62.7% of oocytes reached to cleaved stage and only 11.8% of embryos had good quality.

Borges *et al* reported that artificial oocyte activation can improve ICSI cycles outcomes in epididym (16). Although calcium ionophopre treatment has been widely applied in human oocytes and evidence of calcium ionophopre toxicity on gametes and embryos has not been reported but possible risks may still be exist (16-17). Therefore embryo transfer was not accepted by our center’s Ethic Committee. Nakagawa *et al* reported 84.9% fertilization and 64% cleavage rates after exposure of unfertilized oocytes with calcium ionophore. Also they showed normal chromosomal structure in analyzed oocytes (18).

It is debate about DNA structure of achieved embryos after oocyte activation. These embryos may be resulted from parthenogenesis and sperm DNA may be not contributed in embryonal formation. Lu *et al* explained this debate. After calcium ionophor exposure of unfertilized oocytes, they analyzed achieved embryos and showed seven embryos with 46 XY karyotype. So they concluded that DNA contents of sperm participated in embryonal formation (17). 

Furthermore successful pregnancy and delivery of healthy babies has reported after oocyte activation with calcium ionophor in ICSI cycles (19-21). According to a recent study, combination of calcium ionophore could activate the unfertilized oocytes 20-68 hours after ICSI and the best outcome were achieved after 20 hours (17). So time duration of exposure with calcium ionophore may affect ICSI outcome. 

## Conclusion

According to our results, activation of oocytes with calcium ionophore had an acceptable fertilization rate, however high quality embryos remain low. We propose future studies to evaluate embryo quality and possible teratogenic effects of calcium ionophore on obtained embryos in ART cycles.
